# The Effect of *Saccharomyces cerevisiae* Fermentation Product Supplementation on Pro-Inflammatory Cytokines in Holstein Friesian Cattle Experimentally Inoculated with Digital Dermatitis

**DOI:** 10.3390/ani14223260

**Published:** 2024-11-13

**Authors:** Marlee Henige, Kelly Anklam, Matthew Aviles, Julia Buettner, Summer Henschel, Ilkyu Yoon, Jeffrey Wheeler, George Dawson, Jodi McGill, Dörte Döpfer

**Affiliations:** 1 Department of Medical Sciences, School of Veterinary Medicine, University of Wisconsin-Madison, Madison, WI 53706, USA; kelly.anklam@wisc.edu (K.A.); maviles2@wisc.edu (M.A.); jabuettner@wisc.edu (J.B.); srhenschel@outlook.com (S.H.); dopfer@wisc.edu (D.D.); 2 Diamond V, Cedar Rapids, IA 52404, USA; iyoon@diamondv.com (I.Y.); jgwheeler@diamondv.com (J.W.); george_dawson@diamondv.com (G.D.); 3 Department of Veterinary Microbiology and Preventative Medicine, Iowa State University, Ames, IA 50011, USA; jlmcgill@iastate.edu

**Keywords:** lameness, postbiotic, infection model, pro-inflammatory cytokines, M-stage

## Abstract

Digital dermatitis (DD) poses a major animal welfare concern for the dairy industry, with even broader economic implications for the agricultural industry worldwide. The postbiotic, a *Saccharomyces cerevisiae* fermentation product (SCFP), has had a positive influence on the innate immune system of cattle, which makes it a potential candidate as a feed supplement as part of a prevention strategy for DD. This study investigated the effect of a commercial SCFP feed supplement compared to a control feed supplement on the production of pro-inflammatory cytokines (IL-1β and IL-6) by peripheral blood mononuclear cells (PBMCs) in Holstein Friesian steers experimentally infected with DD. The results showed that SCFP supplementation was associated with an overall reduced IL-1β production (*p* = 0.005), particularly prior to experimental inoculation with a DD lesion homogenate. However, the results of the analysis suggest that the innate immune system in the SCFP group became prepared to respond more rapidly to DD infection post-inoculation. During active (M2), chronic (M4), and focal flare-ups (M4.1) of DD, SCFP supplementation resulted in a more rapid secretion of IL-1β (M2: *p* = 0.038; M4/M4/1: *p* = 0.034). A more rapid response to DD infection for IL-6 was only found for chronic (M4) and focal flare-ups (M4.1) of DD (*p* = 0.006). These findings emphasize the difference in cytokine response between various stages of DD in the SCFP group compared to the control, highlighting implications for DD prevention and treatment.

## Simple Summary

Digital dermatitis (DD) is a painful bacterial disease affecting the feet of cattle worldwide, leading to cases of lameness and decreased production rates. Growing concerns for antimicrobial resistance and antibiotic usage in food-producing animals have created a need for alternative methods of treatment or prevention. Feed supplements have been widely used to boost immune function in various species, making them a potential alternative preventative method for cases of DD. *Saccharomyces cerevisiae* fermentation products (SCFPs), a yeast-derived postbiotic, has been found to improve the immune function of cattle when faced with various diseases. To assess the impact of SCFP supplementation on the immune function of steers with DD, markers of inflammation, pro-inflammatory cytokines, were evaluated. The results found that SCFP supplementation was associated with an overall reduction in specific pro-inflammatory cytokines prior to the development of DD. However, upon the development of DD, SCFP-supplemented steers had a faster pro-inflammatory response to the bacterial infection when compared to steers that did not receive SCFP supplementation. These findings emphasize the effect SCFP supplementation has on the immune response of steers upon the development of DD and highlight the potential implications it has as an alternative preventative strategy.

## 1. Introduction

Digital dermatitis (DD), also commonly referred to as hairy heel warts, is the primary cause of lameness in cattle worldwide. This infectious disease is characterized by painful, circumscribed, ulcerative to papillomatous lesions found on the plantar aspect of the foot on or above the coronary band between the heel bulbs [1]. While there is no prevalence estimate for digital dermatitis on a global scale, multiple estimates have been published for individual countries. These estimates range anywhere from 1.4% to 39%, depending on location, management practices, and preventative measures [2]. Digital dermatitis poses important animal welfare concerns and presents significant economic challenges. Cattle with DD have been associated with decreased production rates in terms of weight gain and milk yield, poor reproductive performance, increased rates of culling, and substantial treatment and prevention costs [3,4,5].

Despite the great economic and welfare challenges posed by DD, many questions remain regarding the etiology, transmission, treatment, and prevention of the disease. Studies investigating the etiology of DD have cultured and sequenced multiple bacterial agents, demonstrating that DD is a multi-bacterial disease. The most consistent isolates from multiple studies have been identified as spirochetes belonging to the genus *Treponema* [6,7,8,9,10].

While these Treponemes have been implicated as the primary contributors to DD, the immune response to such a disease is complex, involving both humoral and cell-mediated pathways [11,12,13,14]. The infection and corresponding tissue damage caused by DD elicit the host to produce elevated levels of pro-inflammatory cytokines, such as interleukin-1β (IL-1β) and interleukin-6 (IL-6), released into the blood stream. These pro-inflammatory cytokines are crucial for the recruitment of immune cells to the site of infection or tissue damage, resulting in inflammation [15,16]. While the presence of IL-1β and IL-6 is necessary to initiate the body’s natural immune response, persistence of these cytokines can lead to chronic inflammation that exacerbates lesion progression and results in prolonged healing [15,16,17]. Understanding the nuanced immunological dynamics in response to DD is essential for the development of targeted therapeutic interventions and improving current cattle health management practices.

Given the rising concerns regarding antimicrobial use in food-producing animals, there is an increasing interest in exploring alternative treatment and preventative measures for infectious diseases. One promising strategy is the supplementation of postbiotic *Saccharomyces cerevisiae* fermentation products (SCFPs). Such supplementation consists of a complex blend of bioactive metabolites, including amino acids, organic acids, polyphenols, lipids, and B vitamins [18,19,20]. Supplementation with SCFP has been shown to positively impact the performance, health, and immunity of cattle, as evidenced by an improved average daily gain, increased bodyweight, and greater milk production [21,22]. SCFP supplementation has also shown potential in enhancing immune function and controlling inflammatory responses of other bacterial diseases in cattle [23,24,25]. The specific SCFP blend used in this study has previously demonstrated immune-modulating capabilities during inflammatory challenges or stressors [26,27,28].

Previously, our group assessed the impact of SCFP supplementation in a commercial facility to evaluate its effectiveness in controlling or preventing DD in lactating dairy cows [29]. The results showed that cows supplemented with SCFP were associated with reduced rates of active DD lesions. Our group also observed protective effects of SCFP against the development of experimentally induced active DD lesions, suggesting that SCFP provides support in reducing the risk of DD infection [30].

This study aims to explore the effect of SCFP supplementation on modulating the innate immune system in lightweight Holstein Friesian (HF) steers experimentally inoculated with DD through assessment of the capacity for peripheral blood mononuclear cells to produce pro-inflammatory cytokine levels when stimulated with toll-like receptor (TLR) agonists. Statistical analysis using Wilcoxon rank sum tests, bivariate analysis, and fixed and random effects regression models was used to generate the most meaningful results for this dataset. We hypothesized that SCFP supplementation has a significant pro-inflammatory effect on the innate immune response, as indicated by greater concentrations of IL-1β and IL-6 upon stimulation of immune cells isolated from whole blood in vitro.

## 2. Materials and Methods

The animal care and management procedures for this study were approved by the University of Wisconsin-Madison Institutional Animal Care and Use Committee (A005762-R03). The study, conducted between June and September 2022 for a total duration of 120 days using 120 Holstein steers, followed a complete randomized design and included three phases: the field phase, the challenge phase, and the transmission phase. The 6-week-long field phase served as a baseline for monitoring cattle health and allowing adaptation to the study diets. During the challenge phase of 6-weeks in duration, a subset of steers from both the control and SCFP groups were subjected to experimental inoculation with DD to evaluate the efficacy of SCFP supplementation on the prevention of DD lesions. The final 5-week transmission phase regrouped the subset of challenge steers with their unchallenged counterparts from the field phase to evaluate the efficacy of SCFP supplementation on the prevention of DD transmission in small populations of cattle.

### 2.1. Experimental Animals

**Figure 1:**
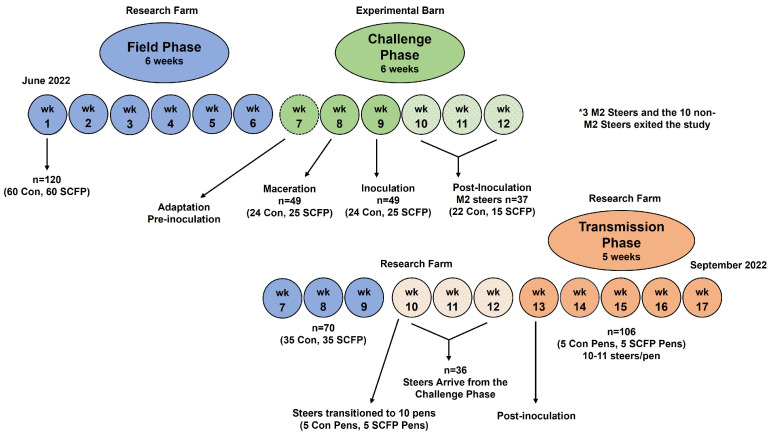
Study timeline for the evaluation of steers supplemented with or without *Saccharomyces cerevisiae* fermentation products utilizing a digital dermatitis experimental infection model. Con = control group; SCFP = SCFP supplemented group.

A total of 120 HF steers, six to seven months of age and weighing between 120 and 200 kg bodyweight (BW), were acquired from three commercial beef farms in Wisconsin (*n* = 95, 15, and 10, respectively). Upon arrival, each steer received visual identification ear tags (ID) and were vaccinated with Bovi-Shield Gold 5 (Zoetis, Kalamazoo, MI, USA). At the study’s start, none of the steers showed any clinical signs of hoof disease. During the field phase, steers were housed in two group pens (60 steers per pen, with approximately 3.3 m^2^ walking space and 2.5 m^2^ lying space per steer) at the University of Wisconsin-Madison research farm that featured a naturally ventilated, open, three-sided barn with cement flooring, straw bedding, and an outside loafing area. During the challenge phase, fifty steers (25 steers per group) were transported to an experimental barn at the University of Wisconsin-Madison that featured an enclosed facility with positive-pressure ventilation. The fifty steers were housed in six pens (each pen measured 40 m^2^, 4.8 m^2^ per steer) with slatted floor surfaces. The pens were cleaned daily with a water hose and automatically flushed six times per day with fresh water. After the challenge phase, the subset of experimentally infected steers with DD lesions was returned to the research farm and reunited with the healthy (non-challenged) steers for the transmission phase of the study. During the transmission phase, healthy and infected steers were co-mingled in groups of 10–11 steers in 10 pens (each pen had approximately 3.3 m^2^ walking space and 2.5 m^2^ lying space per steer). These co-mingled groups of steers were matched by bodyweight but were randomized by the control versus SCFP supplementation. A timeline illustrating the three phases of the study is shown in Figure 1.

### 2.2. Treatments

Steers were randomly split into control and SCFP supplementation groups (n = 60 per group, 1 pen per group). Steers received their respective supplementation throughout the duration of the study, and investigators were blinded as to which groups received the control or SCFP supplementation. The experimental diets were formulated to achieve an approximate average daily gain (ADG) of 1.81 kg/d based on the 2021 NRC guidelines [31]. Pellets containing no supplement (control) or 12 g per day steer NutriTek^®^ (SCFP, Diamond V, Cedar Rapids, IA, USA) were mixed into the corn silage-based total mixed ration (TMR) based on the manufacturer recommendations. Table 1 contains the ingredients and chemical composition of the TMR diet, along with concentrations of both treatment supplementations. The rations were fed without refusals, and the daily dose of supplement was consumed by the steers. During the challenge phase, steers were fed individually in head gates to ensure full consumption of the TRM and supplementation. Steers had ad libitum access to water.

### 2.3. Data Collection

Steers were subject to weekly health exams where bodyweight, rectal temperature, heart rate, and respiratory rate were measured and recorded. Weekly monitoring of DD lesions was performed for all steers and recorded using M-stages on a 5-point scale. Lesions were classified as M0 if no lesion was observed at the coronary band, M1 if a focal lesion < 2 cm in diameter was observed surrounded by healthy skin, M2 if an active lesion ≥ 2 cm in diameter was observed, M4 if the lesion was chronic hyperkeratotic (M4) or proliferative (M4P), and M4.1 if the lesion was chronic hyperkeratotic (M4.1) or proliferative (M4.1P) combined with M1 lesions within the lesion perimeter [32,33]. The most severe DD score per two hindfeet, steer, and timepoint was recorded using the following hierarchy of severity: M0 < M1 < M4.1 < M4.1P < M4 < M4P < M2.

### 2.4. Blood Sample Collection

Whole blood samples (~10 mL) were collected 2 h post-feeding by coccygeal venipuncture in heparinized blood vacutainer tubes (Becton Dickinson, Franklin Lakes, NJ, USA) upon arrival (week 1, n = 43 random steers), prior to inoculation (week 7, n = 40 in 2 random sets of 20 for the SCFP and control groups, respectively), three weeks post-inoculation (week 10, n = 49, 25 for the SCFP and 24 for the control group), and six weeks post-inoculation (week 13, n = 35, 17 for the SCFP and 18 for the control group). These sample sizes represent convenience sampling. All samples were chilled to 4 °C and shipped overnight on ice to Iowa State University.

### 2.5. Proinflammatory Cytokine Testing

Whole blood samples were processed immediately upon arrival to Iowa State University to isolate PBMCs. Cells were isolated from buffy coats by density centrifugation, as previously described [34]. Contaminated red blood cells were removed using hypotonic lysis. Cells were washed twice, counted as cells/mL, and resuspended in complete Roswell Park Memorial Institute (cRPMI) medium composed of RPMI-1640 (Gibco, Carlsbad, CA, USA) supplemented with 2 mM I-glutamine, 25 mM hydroxyethyl piperazineethanesulfonic acid buffer, 1% antibiotic-antimycotic solution, 1% nonessential amino acids, 2% essential amino acids, 1% sodium pyruvate, 50 µM 2-mercaptoethanol (Sigma, St. Louis, MO, USA), and 10% (*v*/*v*) fetal bovine sera (FBS). Blood cells were cryopreserved as follows: Briefly, PBMCs were resuspended at 2 × 10^7^ cells/mL in 1 mL of precooled FBS containing 10% dimethyl sulfoxide (DMSO) and rapidly brought to −80 °C in polystyrene containers, which ensured a slow drop in temperature. After 24 h, the cryovials were transferred to a liquid nitrogen tank and remained there until analysis.

Samples were stimulated using the protocol described by Mahmoud et al. (2020) with some adaptations [27]. Briefly, PBMCs (1 × 10^6^ cells/mL, 1 mL/well) were plated into triplicate 96-well round-bottom plates, and samples were stimulated with toll-like receptor (TLR) agonists of 1 µg/mL lipopolysaccharide (LPS), 10 µg/mL Pam3CSK4 (PAM), or a mixture of 50 µg/mL Poly(I:C) with 10 µg/mL imiquimod (Poly). All stimulants were purchased from InvivoGen (San Diego, CA, USA). Mock samples were cultured with cRPMI medium only. The selection of each TLR stimulant used was chosen based on their varied properties to “mimic” various infectious stimuli. PAM and LPS were used to simulate Gram-positive and Gram-negative bacterial infections, respectively. Poly was used to simulate viral infections [35,36,37]. Samples were cultured for 48 h, and then, cell supernatants were pooled from triplicate wells and frozen at −70 °C until further analysis of the cytokine levels.

The concentration of cytokines in cell culture supernatants was determined using bovine commercial ELISA kits. Bovine IL-1β was measured using a commercial ELISA kit (intra-assay CV 4.6% and inter-assay CV 7.7%), and IL-6 was measured using a commercial ELISA kit (intra-assay CV 4.1% and inter-assay CV 5.0%) from ThermoFisher Scientific (Madison, WI, USA), according to the manufacturer’s instructions. Each sample was assayed in duplicate.

The results from this testing measured the capacity for bovine immune cells to produce pro-inflammatory cytokines IL-1β and IL-6 in response to TLR agonists. This was not a measurement of active IL-1β and IL-6 concentrations in the serum upon blood collection. Hence, the term “cytokine production” implies the readiness of blood cells to produce the cytokines under study upon stimulation.

### 2.6. Statistical Analysis

All statistical analyses were conducted using R version 4.3.0 software [38]. The packages used were lme4 (v1.1-34), lmerTest (v3.1-3), and emmeans (v1.8.6) [39,40,41].

M-stages were combined into the following 5 groups: M0, M1, M2, M4/M4.1, and M4P/M4.1P. Frequencies of the resulting M-stages were calculated and tabulated throughout the study. The time period of detection and sampling was computed as baseline (week 1), pre-inoculation (week 7), and post-inoculation (weeks 10 and 13). The resulting time variable was called a timepoint, and the number of observations per timepoint are shown in Table 2.

Numeric variables for the IL-1β and IL-6 concentrations for each stimulant were generated by adding 1 to all values, log-transforming the values, and calculating the difference in observed values between the stimulant (LPS, PAM, and Poly) versus mock assays. The resulting two outcome variables were the log-transformed differences for IL-1β and IL-6, named log_diff_IL1B and log_diff_IL6, respectively. In addition, bodyweight (kg) was log-transformed for the purpose of statistical analysis, resulting in the variable named log_BW.

Statistical testing for differences between the numeric variables and the outcome variables, log_diff_IL1B and log_diff_IL6, was accomplished using Wilcoxon rank sum tests [42]. Linear regression models with fixed effects and linear mixed regression models with random effects for ID fitted to the intercept were used to quantify the same associations while correcting for potential confounders, repeated measures, and interactions, as described below [43]. The lsmeans differences are the estimates of averages corrected for other covariates in the linear models, while contrasting represents the statistical testing for statistically significant differences between lsmeans values stratified by group [42]. The lsmeans and their 95% confidence intervals were graphed stratified by group and M-stage. In addition, contrasts were tabulated, stratified by group.

The statistical analysis of the dataset was completed in three steps to compare estimates, 95% confidence intervals, and goodness of fit:

(1) A Wilcoxon rank sum test was performed for the pairs of log-transformed cytokine difference values stratified by group.
(2) Linear regression models for the outcome variables with fixed effects only including lsmeans and contrasts.
(3) Linear mixed regression models for the outcome variables with random effects for steer ID fitted on the intercept including lsmeans and contrasts.

During step (1), descriptive analysis included calculating the means and standard deviations stratified by group, as shown in Table 3 and Table 4. In Table 5, statistically significant differences of pairwise raw averages at the 95% confidence level without correction for confounding or interaction with variables, among which were log-transformed bodyweight (log_BW), M-stage, and timepoint, were derived from the Wilcoxon rank sum test [42].

The full regression equations for steps (2) and (3) are shown in Equations (1) and (2). The final linear mixed regression models created in steps (2) and (3) were reached using backwards step elimination. Interaction terms were retained if the association with the outcome variable was statistically significant at the 95% confidence level [43].

Equation (1): Full linear regression model with fixed effects only.

y ~ intercept + Group + Timepoint + log_BW + M-stage + Group*Timepoint + Group*M-stage + error(1)

y = log-transformed IL-1β or IL-6 concentration for stimulant (LPS, PAM, or Poly) versus mock; Group = supplementation group (control versus SCFP supplementation); Timepoint = time of blood sample collection (baseline, pre-inoculation, or post-inoculation); log_BW = log-transformed bodyweight (kg); M-stage = M-stage of current DD lesion.

Interaction terms: Group*Timepoint and Group*M-stage.

Equation (2): Full linear regression model with steer ID as a random effect.

y ~ intercept + Group + Timepoint + log_BW + M-stage + Group*Timepoint + Group*M-stage + 1|ID + error(2)

y = log-transformed IL-1β or IL-6 concentration for stimulant (LPS, PAM, or Poly) versus mock; Group = supplementation group (control versus SCFP supplementation); Timepoint = time of blood sample collection (baseline, pre-inoculation, or post-inoculation); log_BW = log-transformed bodyweight (kg); M-stage = M-stage of current DD lesion; ID = steer identification number.

Interaction terms: Group*Timepoint and Group*M-stage.

## 3. Results

### 3.1. Clinical Evaluation

Prior to inoculation, during the field phase, no DD lesions were observed on any of the steers enrolled in the study. During the challenge phase, the hind feet of the steers were clinically evaluated for DD lesions using visual inspection in the chute, which is currently considered the gold standard for diagnosis. Seven days post-inoculation, the steers’ hind feet were inspected for lesion development. Experimentally induced DD lesions were observed above the heel bulbs and below the dew claws. As reported by Anklam et al., 2024, all steers in the challenge phase developed either a M1 or M2 lesion on at least one foot post-inoculation, but the control steers had significantly more M2 lesions compared to the SCFP steers [30]. Table 2 summarizes the DD lesion status of the steers at the blood collection timepoints during the study while stratified by group. Table 3 summarizes the bodyweights per phase of the study.

### 3.2. Cytokine Evaluation

The pro-inflammatory cytokine testing performed in this study analyzed the capacity for bovine immune cells to produce pro-inflammatory cytokines (IL-1β and IL-6) in response to stimulation with TLR agonists. Table 4 displays the raw average pro-inflammatory cytokine levels for IL-1β and IL-6 in response to the stimulation of innate immune cells from steers supplemented with and without SCFP for the following blood collection timepoints: baseline (week 1), pre-inoculation (week 7), and post-inoculation (weeks 10 and 13).

### 3.3. Bivariate Analysis

The bivariate analysis was conducted using Wilcoxon rank sum testing to evaluate the differences in IL-1β and IL-6 production upon the stimulation of innate immune cells from steers supplemented with and without SCFP for each blood collection timepoint throughout the study. The results of this testing revealed a statistically significant difference of IL-1β production levels between supplementation groups during the pre-inoculation timepoint (week 7) upon stimulation with LPS (Table 5). No statistically significant differences in IL-6 production were found.

### 3.4. Linear Regression Analysis

Only models with statistically significant findings at the 95% confidence level are reported in Table 6, Table 7, Table 8, Table 9 and Table 10. Linear regression models with fixed effects only are reported if a statistically significant association was found in relation to the group. Table 6 displays the final linear regression model with fixed effects only that quantified associations between IL-6 production as a consequence of PAM stimulation, group, timepoint, log_BW, and the interaction term Group*M-stage. The results of this analysis revealed a statistically significant (*p* = 0.018) increase in IL-6 production when the bodyweight of steers increased. The interaction of group and M-stage was statistically significant (*p* = 0.034) and interpreted as follows: within the SCFP group compared to the control group, when a transition from M0 to M4/M4.1 occurred, the increase in IL-6 production was statistically significantly more rapid.

Table 7 exhibits the final linear mixed regression model with random effects for steer ID fitted on the intercept that quantifies associations between the IL-1β response to LPS stimulation, group, timepoint, log_BW, and the interaction term Group*M-stage. The results of this analysis revealed a statistically significant (*p* = 0.005) reduction in IL-1β production of SCFP-supplemented steers when compared to the control group. The interaction between group and M-stage was statistically significant (*p* < 0.04), indicating a statistically faster increase in IL-1β production in SCFP-supplemented steers when transitioning from M0 to either M2 or M4/M4.1 lesions compared to the control steers (M2: *p* = 0.034; M4/M4.1: *p* = 0.038).

The final linear mixed regression model with random effects for steer ID fitted on the intercept that quantifies associations between the IL-1β response to Poly stimulation, group, timepoint, and log_BW is displayed in Table 8. The results of this analysis revealed a statistically significant (*p* = 0.026) reduction in IL-1β production at the pre-inoculation timepoint (week 7) when compared to baseline (week 1). There was also a statistically significant (*p* = 0.021) increase in IL-1β production at the post-inoculation timepoint (weeks 10 and 13) when compared to baseline (week 1).

Table 9 presents the final linear mixed regression model with random effects for steer ID fitted on the intercept that quantifies associations between the IL-6 response to PAM stimulation, group, timepoint, log_BW, and the interaction term Group*M-stage. The results of this analysis revealed a statistically significant (*p* = 0.006) interaction between group and M-stage, indicating a significantly faster increase in IL-6 production in SCFP-supplemented steers when transitioning from M0 to M4/M4.1 lesions compared to the control steers.

The final linear mixed regression model with random effects for steer ID fitted on the intercept that quantifies associations between the IL-6 response to Poly stimulation, group, timepoint, and log_BW is shown in Table 10. The results of this analysis revealed a statistically significant (*p* = 0.029) increase in IL-6 production of steers with increased bodyweight.

### 3.5. Lsmeans and Contrasting Analysis

Lsmeans values were calculated for each final model from steps (2) and (3) to estimate the average pro-inflammatory cytokine production per group while correcting for other covariates in the linear models. Contrasts of these values were then analyzed for statistically significant differences stratified by group.

**Figure 2:**
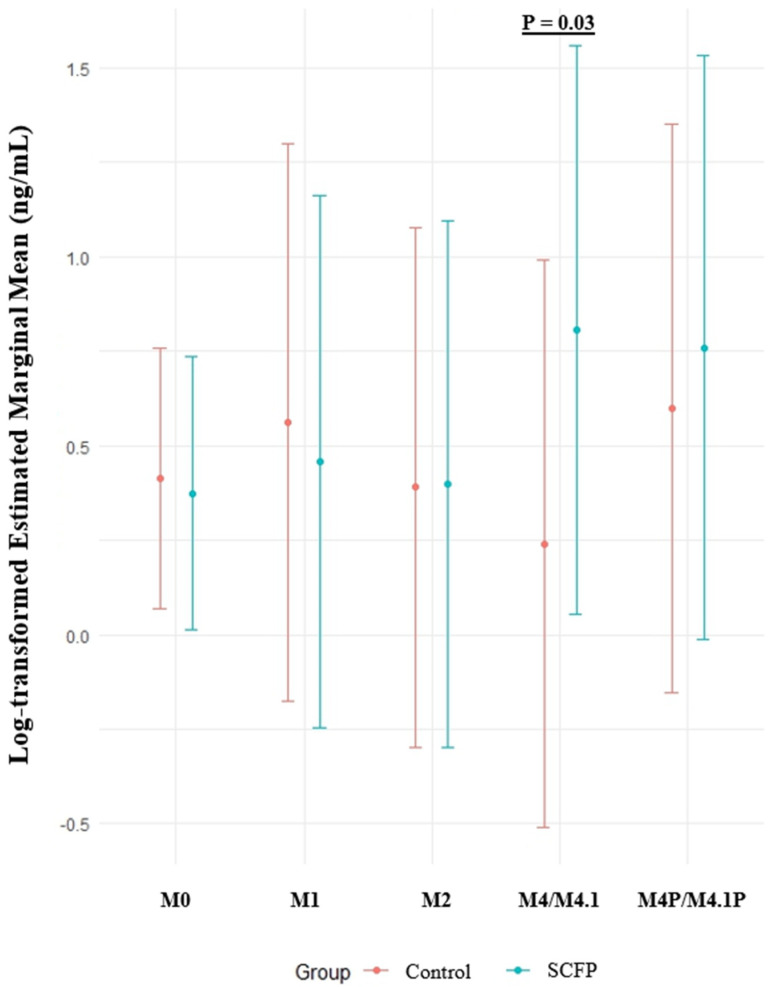
The log-transformed estimated marginal mean IL-6 cytokine production (ng/mL) upon stimulation with PAM (Pam3CSK4) by innate cells from the whole blood of steers supplemented with or without *Saccharomyces cerevisiae* fermentation products (SCFPs) stratified by M-stage of digital dermatitis lesions. N = 167.

The results for the fixed effects model, which showed statistically significant associations (*p* < 0.05) between the supplementation group and IL-6 production upon PAM stimulation, are presented in Table 6. A visual depiction of the least square means (lsmeans) and corresponding *p*-values from the contrasts for this model is shown in Figure 2. As illustrated, there was a statistically significant difference in the contrasts of lsmeans between the supplementation groups of steers with M4/M4.1 lesions compared to M0.

None of the random effects models presented in Table 7, Table 8, Table 9 and Table 10 showed statistically significant contrasts in IL-1β or IL-6 production between the SCFP and control supplementation groups.

## 4. Discussion

### 4.1. Sample Size and Significance Level

The analysis of estimated marginal means for IL-6 production upon stimulation with PAM illustrated in Figure 2 had notably large 95% confidence intervals. This was likely due to the limited size of our dataset and large variance between individual steers. Associations were interpreted only if they reached statistical significance at the 95% confidence level. No interpretation was provided for trends in statistical significance at *p* < 0.1 [43]. This sample size represents convenience sampling, and further studies should be conducted with an increased sample size. Cautious model selection resulted in reporting statistically significant associations only at the 95% confidence level to prevent false-positive results.

### 4.2. Wilcoxon Rank Sum Testing

The Wilcoxon rank sum test, also known as the Mann–Whitney Test, is a nonparametric test used for two independent samples to test whether the mean values for each group are different while accounting for non-normally distributed data [42]. The Wilcoxon rank sum test used the groups to test for differences in the mean pro-inflammatory cytokine levels between the control and SCFP-supplemented steers without correcting for potential confounders, among which include bodyweight, timepoint, M-stage, and steer ID (Table 4). A statistically significant difference in IL-1β levels was revealed during the pre-inoculation timepoint (week 7). This demonstrates that SCFP supplementation had an immunomodulatory effect on steers from the start of supplementation until just prior to experimental inoculation with DD. This difference was only noted upon stimulation with LPS, which is used to mimic Gram-negative bacterial infections [35]. Treponema species, which are believed to be primary contributors to the pathogenesis of DD, are Gram-negative bacteria. Therefore, SCFP supplementation was interpreted as a potential preventative measure not only against DD but also against other Gram-negative bacterial infections of bovine digital skin. To further characterize the differences in IL-1β concentrations between supplementation groups and to account for potential confounding factors, linear regression model analysis was performed (Equations (1) and (2)). The Wilcoxon rank sum test results were not interpretable, since the test results were confounded by bodyweight, timepoint, M-stage, and steer ID.

### 4.3. Comparing Fixed and Random Effects Models

To account for possible confounding factors and to analyze the direction of any differences in the pro-inflammatory cytokine levels, linear regression models were developed for the difference in stimulant versus the mock assay for both IL-1β and IL-6 (Equations (1) and (2)). The initial linear regression models included fixed effects only (Equation (1)). Fixed effects models are commonly used in linear regression analysis for their simplicity and ability to account for unobserved sources of bias in the estimation of model parameters [42,43]. While these models can provide accurate estimates of model parameters, individual steer cytokine levels need to be considered.

Unlike fixed effects models, random effects models account for variability within individual observations across a broader group [42,43]. To account for the variance in individual steer cytokine levels, linear mixed regression models with a random effect for steer ID fitted to the intercept were generated (Equation (2)). By analyzing the characteristics of individual steers using random effects models, instead of pooling data together, as with fixed effects models, the model incorporates more variance, leading to more accurate estimates of variance, standard errors, and confidence intervals for effect estimates [42,43].

Given the relatively limited dataset and the use of convenience sampling for blood collection, the analysis should account for the individual characteristics of each steer. Based on these conclusions, further analysis and interpretation of the random effects models (Equation (2)) was preferred to interpret the fixed effects models.

### 4.4. Effect of SCFP Supplementation by Stimulant

Immunologic studies use different stimulants to mimic a variety of infections. LPS and PAM are commonly used to stimulate Gram-negative and Gram-positive bacterial infections, respectively [35,37]. In contrast, Poly is used to simulate viral infections [36]. The results from the analysis of each stimulant used in our study can provide insights into the immunomodulatory effects of SCFP supplementation upon in vitro host cell exposure to bacterial (Gram-negative or Gram-positive) or viral infection.

The results of the initial Wilcoxon rank sum tests provided evidence of immunomodulation by SCFP in relation to IL-1β pathways associated with Gram-negative bacterial infection. This was further evident in the linear mixed regression model with random steer ID effects investigating IL-1β production in response to LPS stimulation (Table 6). The results of this random effects regression model revealed an overall reduction in the IL-1β concentrations from stimulated cells of steers supplemented with SCFP. This suggested that SCFP supplementation had an immunomodulatory effect that was associated with decreased IL-1β production. This model also revealed that SCFP-supplemented steers exhibited a faster IL-1β response when transitioning from M0 to M2 or from M0 to M4/M4.1 lesions compared to the control steers. This immunomodulatory effect of SCFP supplementation in response to LPS stimulation suggests SCFP supplementation is a potential preventative measure against DD, as well as other Gram-negative bacterial infections of bovine digital skin. However, the contrast testing did not reveal any significant differences in lsmeans averages of IL-1β between supplementation groups. A detailed analysis of the data for active (M2) or chronic (M4.M4.1) DD lesions (Table 7) suggests that the SCFP group exhibits a more rapid IL-1β response to infection with Gram-negative bacteria, as simulated by LPS stimulation. This increased speed of inflammatory response could be associated with “trained Immunity”. Trained immunity is a phenomenon where the stimulation of innate immune cells, especially macrophage precursors in the bone marrow, can result in an enhanced innate response and resistance to infections [44,45]. In addition, active IL-1β is released after inflammasome activation, which might enhance inflammation, leading to better pathogen control or immunopathology. This phenomenon implied that innate defense cells could have been primed for inflammasome activation after SCFP treatment [46,47]. There were no statistically significant effects of LPS stimulation upon the IL-6 levels.

A similar study looking at the effect of SCFP supplementation on neonatal calves challenged with Bovine Respiratory Syncytial Virus (BRSV) found that LPS stimulation of PBMCs did not have an effect on IL-1β production prior to the BRSV challenge. This study instead found an association between SCFP-treated calves and increased IL-6 production prior to the BRVS challenge up LPS stimulation [27]. This contradiction in findings can be correlated to the age of the cattle used in the study. IL-1β concentrations are known to be elevated in neonatal calves during the first week of life but consequently drop off during the second week of life [48]. This downward transition in concentration could account for the apparent lack of change seen in IL-1β levels during the study. In contrast, IL-6 remains more elevated during the first three weeks of life in calves than IL-1β [48]. With these levels already at a relatively increased concentration, stimulation with LPS would only further increase their concentration, thus aligning with the results of the study. Burdick Sanchez et al. (2020) also looked at the effect of SCFP supplementation, particularly in weaned beef calves upon a LPS challenge. The results of their study found only a trend for association between SCFP calves and reduced IL-6 levels [49]. Samples, however, were only analyzed 2 h prior to the LPS challenge up to 24 h post-challenge, which is a relatively short timeframe of study. This reduction in IL-6 was attributed to the significantly reduced levels of TNF-α in SCFP-supplemented calves, as TNF-α stimulates the release of IL-6 [49]. In a similar study, by Klopp et al. (2022), that evaluated SCFP supplementation in neonatal diary calves upon LPS challenge, no differences in IL-6 production were observed. The samples were again only analyzed 2 h prior to the LPS challenge up to 24 h post-challenge [50]. The relatively short study timeframe and focus on the post-challenge effects of both the Burdick Sanchez and Klopp studies do not provide evidence for the possible effects of SCFP supplementation prior to disease challenges. They do, however, provide support that SCFP supplementation does not inhibit the host’s natural immune response to inflammatory stimuli. The findings from these additional studies highlight the complex balance of innate immune cells, particularly pro-inflammatory cytokines, during cattle’s lifetime that plays an important role in the ability for SCFP supplementation to enhance normal innate immune responses. Future studies should focus on pro-inflammatory cytokine responses with regards to magnitude, velocity of change, and duration for IL-1β upon exposure to different M-stages using increased sample sizes or frequency of sampling.

The initial bivariate analysis evaluating PAM stimulation upon cytokine production found no differences between supplementation groups (Table 5). In contrast, the linear regression model with fixed effects only in step (2) and the linear mixed regression model with random effects in step (3) revealed an association between the interaction of SCFP*M4/M4.1 lesions and IL-6 (Table 6 and Table 9). This was interpreted as follows: upon transition from M0 to M4/M4.1, the SCFP group had a statistically significantly faster IL-6 response compared to the control group. Since M4/M4.1 lesions are more chronic, having a faster downregulation of the inflammatory response, as indicated by IL-6, is advantageous [51,52]. This provides evidence that SCFP supplementation has an immunomodulatory effect that stimulates IL-6 production in response to Gram-positive bacteria. While Gram-positive bacteria are not believed to be the primary cause of DD, such bacteria may still play a role during the initial infection or become introduced as the secondary bacterial infection of a chronic DD lesion. Finding a faster IL-6 response during the chronic stages of DD suggested that SCFP supplementation played a potential role in reactions to chronic DD, including M4 and M4.1 lesions. There was no statistically significant effect of PAM stimulation on the IL-1β levels. The contrasting of the lsmeans for the IL-6 levels showed statistically significant differences for linear regression with fixed effects only (Figure 2) but no statistically significant differences for the linear mixed regression model with random effects for steer ID in step (3) of the analysis. This emphasizes the importance of correcting for individual steer levels of IL-6 to avoid misinterpretation of the results.

The study conducted by Mahmoud et al. (2020) on the SCFP effect in neonatal calves challenged with BRSV, as mentioned above, also found an association between SCFP-supplemented calves and increased IL-6 upon stimulation with PAM prior to the BRSV challenge but no change in IL-1β [27]. While these findings support the results of this study, they can likely be attributed to the natural fluctuation in pro-inflammatory cytokines during the first few weeks of life in calves. This again highlights the complexity of pro-inflammatory cytokines, the host’s natural immune response based on age, and SCFP supplementation that requires further research. Another study looking at the effect of SCFP supplementation on the *Streptococcus uberis* mastitis challenge in mid-lactation dairy cows found no change in IL-1β or IL-6 concentrations from the time of the *Streptococcus uberis* challenge up to 9 days post-challenge [25]. Seeing no change in the pro-inflammatory post-disease challenge supports that SCFP supplementation may have an effect on the innate immune system prior to an inflammatory trigger but does not inhibit the host’s natural immune responses. To gather more information on the effect of postbiotic SCFP supplementation in response to Gram-positive based infections, further studies need to be conducted.

While DD is assumed to be caused by mostly bacterial infection, our analysis of the stimulant Poly was used to investigate SCFP’s effect on simulated viral infections, which may be a random finding. The linear mixed regression model with random effects evaluating IL-1β levels revealed statistically significant differences based on the timepoint at which the blood samples were collected. During the pre-inoculation timepoint, there was a statistically significant reduction in the IL-1β levels. In contrast, there was an elevation in the IL-1β levels during the post-inoculation timepoints (Table 8). Although this is not a group effect comparing the control and SCFP supplementation, this illustrates the body’s natural response to combating infection. Immune pathways are not triggered until the host has been exposed to a source of infection. Once exposed, these pathways are stimulated to upregulate pro-inflammatory cytokine production [15,16,17]. Further analysis of the data assessing IL-6 production in response to Poly stimulation, as shown in Table 9, revealed an association between increased bodyweight and elevated IL-6 levels. This association may be influenced by increasing age, which indicates that a more developed immune system responds more robustly to infection. Both the IL-1β and IL-6 models showed that SCFP supplementation had no statistically significant association between Poly stimulation and cytokine production when stratified by supplementation group, which is in contrast to a previous work on pneumonia in calves where a primary viral infection was followed by a bacterial infection [28].

Mahmoud et al. (2020) similarly found no treatment effect for IL-1β or IL-6 production upon stimulation with Poly when SCFP-supplemented neonatal calves were challenged with BRSV [27]. This evidence further suggests that SCFP supplementation may not play a strong role in “priming” the immune system for challenges against viral infection; however, further studies should be conducted to evaluate SCFP supplementation in response to viral infection.

### 4.5. Effect of SCFP Supplementation over Time

As shown in Table 5, which presents the results of the Wilcoxon rank sum testing, SCFP supplementation exhibited varying associations with pro-inflammatory cytokine concentrations across the different timepoints when the blood samples were collected. The baseline timepoint (week 1) was taken just prior to each group being started on their respective supplementation diets; thus, no difference would be expected between the two groups. The pre-inoculation timepoint (week 7) was taken just prior to the subset of challenged steers being experimentally inoculated with DD. After these weeks of supplementation, there was a statistically significant association between SCFP-supplemented steers and elevated IL-1β concentrations. Supplementation with SCFP could be associated with immunomodulatory effects on IL-1β production, but this association could be confounded by other covariates in the dataset. Wilcoxon rank sum testing does not evaluate the direction of this difference, but the linear mixed regression model with random effects (Table 7) shows this to be a reduction in IL-1β concentration. After correcting for the steer ID, bodyweight, and M-stage during step (3) of the analysis, evaluation of the post-inoculation timepoints (weeks 10 and 13) found no statistically significant differences in the pro-inflammatory cytokine levels, IL-1β and IL-6, between the SCFP and control groups. This emphasizes the necessity to adjust the analysis for confounders within steer ID and clustering within the dataset to ensure accurate interpretation of the results. These results demonstrated that, prior to DD infection, SCFP supplementation resulted in downregulated IL-1β production. However, upon infection with DD and up to 6 weeks later, this reduction in IL-1β production was not confirmed after appropriate correction for confounders and intra-class correlations.

The study of SCFP effects in neonatal calves challenged with BRSV also looked at the effect of time on the results of the pro-inflammatory cytokine levels. They found that both IL-1β and IL-6 concentrations had significant variations during the first 19 days prior to the BRSV challenge but no change from the initial BRSV challenge and up to 10 days post [27]. While these findings provide further evidence that SCFP supplementation may play a role in immunomodulation prior to a disease challenge, this could be confounded by the natural extreme fluctuations of immune cells in neonatal calves and requires further investigation [48]. The Burdick Sanchez et al. (2020) and Klopp et al. (2022) studies did not look at the IL-1β concentrations, but upon investigation of the IL-6 concentrations, found no treatment by time interactions [49,50]. This is likely due to the short timeframe of the study that was mainly focused on the post-challenge effects of SCFP supplementation. Further studies should be conducted to evaluate SCFP supplementation over extended periods of time to evaluate the long-term effects upon cytokine production and other markers of inflammation such as TNFα and acute phase proteins.

### 4.6. SCFP Supplementation and Prevention of Digital Dermatitis

**Figure 3:**
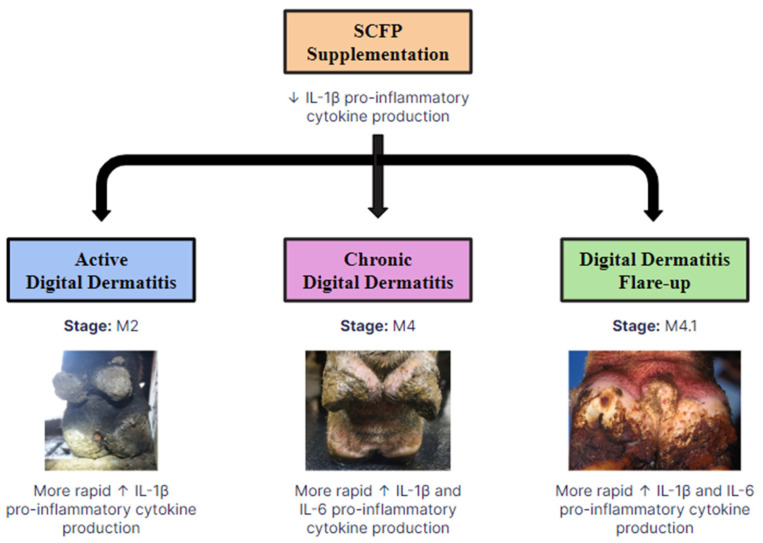
Proposed flowchart for the effects of SCFP treatment on immune function in cases of digital dermatitis (DD). The results of our study suggested that SCFP supplementation differentially modulates the immune function in cattle experimentally inoculated with DD, depending on the stage of disease. During active (M-stage: M2) stages of DD, SCFP supplementation resulted in a more rapid response of the innate immune system to secrete pro-inflammatory cytokine IL-1β, as tested in vitro. This suggested that the innate immune system was primed and became more prepared to robustly respond to DD infection. During chronic (M-stage: M4) and focal flare-ups (M-stage: M4.1) of DD, SCFP supplementation resulted in a more rapid response of the innate immune system to secrete pro-inflammatory cytokines IL-1β and IL-6, as tested in vitro. This suggested an enhancement of the innate regulatory mechanisms of the immune system to better respond to chronic lesions and secondary flare-ups of DD.

Based on the results outlined in this study, SCFP supplementation has promise for the prevention of bovine DD infection. Figure 3 illustrates the interpretation of the results from the data analysis related to the M-stages of DD. Before the DD challenge, there was a decrease in IL-1β concentrations in the SCFP group. On the other hand, the results suggested that the innate immune system in the SCFP group became prepared to respond more rapidly to DD infection post-inoculation. Particularly during active (M2), chronic (M4), and focal flare-ups (M4.1) of DD, SCFP supplementation led to a more rapid response of the pro-inflammatory cytokine IL-1β, as indicated by stimulation of the innate immune system in vitro. The more rapid response to DD infection for IL-6 was only found for chronic (M4) lesions and focal flare-ups (M4.1). These findings emphasize that there is a difference in the cytokine response between active tissue destruction and chronic tissue alterations in the SCFP group compared to the control group, which has implications for the prevention and treatment of DD.

## 5. Conclusions

In this study, the SCFP supplement appears to be a candidate for the prevention of DD in cattle that could be incorporated into strategies aimed at the prevention and control of DD at the herd level. As hypothesized, a pro-inflammatory effect of SCFP supplementation was found after experimental inoculation with DD. In addition, SCFP-supplemented steers showed a more rapid pro-inflammatory cytokine response to DD. Larger sample sizes and in-depth studies of pro-inflammatory cytokines and other messengers of inflammation should be included in future efforts to immunomodulate the acute and long-standing inflammatory reactions during DD, particularly as they relate to chronic DD. The concepts of “trained Immunity” and the inflammasome should also be incorporated into future studies about DD related to inflammation during the pathogenesis of DD.

## Figures and Tables

**Table 1:** Ingredients and chemical composition of the total mixed ration (TMR) for steers supplemented with or without *Saccharomyces cerevisiae* fermentation products (SCFPs ^1^).

**Ingredient, As Fed** **Amount/Animal/day**	**DM %**	**Field Phase and** **Transmission Phase** **Analyzed Result** **kg**	**Challenge Phase** **Analyzed Result** **kg**
Corn Silage 1	36.65	9.09	-
Corn Silage 2	35.70	-	9.53
Corn-Cracked	95.50	2.14	2.14
Soybean Meal	87.90	1.21	1.21
Control Pellet ^2^ or SCFP Pellet ^3^	92.12	0.34	0.34
Amount/Animal (As Fed)		12.79	13.22
Amount/Animal (DM)		6.75	6.82
**Nutrient, Dry Matter (DM)** **Concentration**	**Unit**	**Field Phase and** **Transmission Phase** **Analyzed Result**	**Challenge Phase** **Analyzed Result**
Crude Protein (CP)	%	15.71	15.96
Rumen Degradable Protein (RDP)	%	10.32	10.51
Acid Detergent Fiber (ADF)	%	12.03	12.07
Neutral Detergent Fiber (NDF)	%	21.64	22.71
Non-fiber Carbohydrate (NFC)	%	53.64	52.55
Forage NDF	%	17.29	18.41
Adjusted Total Starch	%	39.80	40.61
Fat	%	2.92	2.88
Calcium (Ca)	%	0.62	0.66
Phosphorus (P)	%	0.39	0.40
Magnesium	%	0.22	0.22
Potassium	%	1.16	1.10
Sulfur	%	0.18	0.18
Sodium	%	0.07	0.07
Chloride	%	0.25	0.25
Added Manganese	mg/kg	36.51	36.14
Added Zinc	mg/kg	35.76	35.40
Added Copper	mg/kg	5.08	5.03
Added Selenium	mg/kg	0.22	0.22
Added Cobalt	mg/kg	0.46	0.46
Added Iodine	mg/kg	0.37	0.37
Vitamin A Add	IU/g	2.97	2.94
Vitamin D Add	IU/g	1.24	1.24
Vitamin E Add	IU/kg	23.57	23.34
Monensin	g/ton	20.69	20.49

^1^ SCFP—NutriTek^®^ (Diamond V, Cedar Rapids, IA, USA). ^2^ Basal pellets with no supplement. ^3^ Basal pellets supplementing 12 g per day and steer of NutriTek.

**Table 2:** Summary of the digital dermatitis lesion status by M-stage ^1^ classification for steers supplemented with or without *Saccharomyces cerevisiae* fermentation products (SCFPs ^2^) at the blood collection timepoints of the study.

Group	Timepoint ^3^	Sample Number	M0	M1	M2	M4/ M4.1	M4P/ M4.1P
Control	Overall	82	41	8	19	7	7
	Baseline	20	20	0	0	0	0
	Pre-inoculation	20	20	0	0	0	0
	Post-inoculation	42	1	8	19	7	7
SCFP	Overall	85	43	13	16	7	6
	Baseline	23	23	0	0	0	0
	Pre-inoculation	20	20	0	0	0	0
	Post-inoculation	42	0	13	16	7	6
Overall		167	84	21	35	14	13

^1^ M-stage definitions: M0—cattle with normal digital skin; M1—lesions < 2 cm in diameter surrounded by healthy skin; M2—active ulcerative or granulomatous lesions ≥ 2 cm in diameter; M4/M4.1 includes M4—chronic lesions characterized by a thickened epithelium and M4.1—lesions < 2 cm in diameter embedded in a circumscribed dyskeratotic skin alteration; M4P/M4.1P includes M4P—chronic lesions characterized by proliferative growth of the epithelium and M4.1P—lesions < 2 cm in diameter embedded proliferative skin alteration. ^2^ SCFP—NutriTek^®^ (Diamond V, Cedar Rapids, IA, USA). ^3^ Timepoint—blood collection timepoint: baseline—week 1; pre-inoculation (week 7), and post-inoculation (weeks 10 and 13).

**Table 3:** The average bodyweight of steers supplemented with or without *Saccharomyces cerevisiae* fermentation products (SCFPs ^1^) at the blood collection timepoints of the study.

	Timepoint ^3^	Sample Number	Control		SCFP		Overall	
			Average	(SD)	Average	(SD)	Average	(SD)
BW ^2^ (kg)	Overall	167 (Con 82, SCFP 85)	217.3	43.3	221.0	43.5	219.2	43.3
	Baseline	43 (Con 20, SCFP 23)	163.8	11.9	169.2	12.9	166.7	12.6
	Pre-inoculation	40 (Con 20, SCFP 20)	213.6	30.3	213.5	28.4	213.6	29.0
	Post-inoculation	84 (Con 42, SCFP 42)	244.5	32.5	252.9	28.8	248.7	30.8

^1^ SCFP—NutriTek^®^ (Diamond V, Cedar Rapids, IA, USA). ^2^ BW—average bodyweight. ^3^ Timepoint—baseline (week 1); pre-inoculation (week 7); post-inoculation (weeks 10 and 13).

**Table 4:** Stimulant-induced IL-1β and IL-6 inflammatory cytokine production (ng/mL) by innate cells from blood collected at three timepoints (baseline, pre-inoculation, and post-inoculation) from steers supplemented with or without *Saccharomyces cerevisiae* fermentation products (SCFPs ^1^).

Pro-Inflammatory Cytokine and Stimulant ^2^	Timepoint ^3^	Sample Number	Control		SCFP		Overall	
			Average	(SD)	Average	(SD)	Average	(SD)
IL-1β with Mock	Overall	167 (Con 82, SCFP 85)	0.003	0.021	0.003	0.020	0.003	0.020
	Baseline	43 (Con 20, SCFP 23)	0.007	0.031	0.011	0.038	0.009	0.034
	Pre-inoculation	40 (Con 20, SCFP 20)	0.000	0.000	0.000	0.000	0.000	0.000
	Post-inoculation	84 (Con 42, SCFP 42)	0.003	0.020	0.000	0.000	0.002	0.014
IL-6 with Mock	Overall	167 (Con 82, SCFP 85)	0.073	0.242	0.056	0.198	0.065	0.220
	Baseline	43 (Con 20, SCFP 23)	0.053	0.166	0.019	0.093	0.035	0.132
	Pre-inoculation	40 (Con 20, SCFP 20)	0.044	0.094	0.009	0.039	0.026	0.073
	Post-inoculation	84 (Con 42, SCFP 42)	0.097	0.312	0.099	0.266	0.098	0.288
IL-1β with LPS	Overall	167 (Con 82, SCFP 85)	0.413	0.363	0.329	0.321	0.370	0.344
	Baseline	43 (Con 20, SCFP 23)	0.527	0.435	0.385	0.374	0.451	0.405
	Pre-inoculation	40 (Con 20, SCFP 20)	0.482	0.392	0.320	0.387	0.401	0.393
	Post-inoculation	84 (Con 42, SCFP 42)	0.326	0.291	0.302	0.255	0.314	0.272
IL-6 with LPS	Overall	167 (Con 82, SCFP 85)	1.776	1.832	1.747	2.405	1.762	2.136
	Baseline	43 (Con 20, SCFP 23)	1.331	0.959	1.265	1.193	1.296	1.078
	Pre-inoculation	40 (Con 20, SCFP 20)	2.367	2.473	2.640	3.601	2.504	3.052
	Post-inoculation	84 (Con 42, SCFP 42)	1.707	1.760	1.587	2.129	1.647	1.942
IL-1β with PAM	Overall	167 (Con 82, SCFP 85)	0.058	0.099	0.050	0.020	0.054	0.127
	Baseline	43 (Con 20, SCFP 23)	0.072	0.101	0.055	0.149	0.063	0.127
	Pre-inoculation	40 (Con 20, SCFP 20)	0.064	0.132	0.094	0.240	0.079	0.192
	Post-inoculation	84 (Con 42, SCFP 42)	0.048	0.081	0.026	0.075	0.037	0.078
IL-6 with PAM	Overall	167 (Con 82, SCFP 85)	0.646	0.956	0.927	0.198	0.789	1.581
	Baseline	43 (Con 20, SCFP 23)	0.426	0.567	0.323	0.534	0.371	0.545
	Pre-inoculation	40 (Con 20, SCFP 20)	1.134	1.028	1.324	2.103	1.229	1.636
	Post-inoculation	84 (Con 42, SCFP 42)	0.518	1.006	1.068	2.393	0.793	1.845
IL-1β with Poly	Overall	167 (Con 82, SCFP 85)	5.020	7.511	4.302	3.551	4.654	5.834
	Baseline	43 (Con 20, SCFP 23)	3.234	1.790	3.022	1.263	3.121	1.516
	Pre-inoculation	40 (Con 20, SCFP 20)	2.810	1.836	1.940	1.370	2.375	1.658
	Post-inoculation	84 (Con 42, SCFP 42)	6.922	10.037	6.128	4.129	6.525	7.639
IL-6 with Poly	Overall	167 (Con 82, SCFP 85)	1.151	2.532	0.824	1.047	0.984	1.926
	Baseline	43 (Con 20, SCFP 23)	0.202	0.298	0.137	0.321	0.168	0.308
	Pre-inoculation	40 (Con 20, SCFP 20)	0.399	0.417	0.428	0.566	0.413	0.491
	Post-inoculation	84 (Con 42, SCFP 42)	1.960	3.341	1.388	2.393	1.674	2.505

^1^ SCFP—NutriTek^®^ (Diamond V, Cedar Rapids, IA, USA). ^2^ Stimulants—mock (negative control); LPS (lipopolysaccharide; mimics Gram-negative); PAM (Pam3CSK4; mimics Gram-positive); Poly (mixture of Poly(I:C) and imiquimod; mimics virus). ^3^ Timepoint—baseline (week 1); pre-inoculation (week 7); post-inoculation (weeks 10 and 13).

**Table 5:** Results of Wilcoxon rank sum testing of steers supplemented with or without *Saccharomyces cerevisiae* fermentation products (SCFPs ^1^) at the blood collection timepoints of the study.

	Stimulant ^2^	Timepoint ^3^	W-Statistic	*p*-Value
IL-1β	LPS	Baseline	279	0.237
		Pre-inoculation	274	0.047
		Post-inoculation	944	0.582
	PAM	Baseline	279	0.135
		Pre-inoculation	194	0.839
		Post-inoculation	1016	0.089
	Poly	Baseline	235	0.914
		Pre-inoculation	268	0.068
		Post-inoculation	862	0.862
IL-6	LPS	Baseline	237	0.874
		Pre-inoculation	196.5	0.935
		Post-inoculation	957	0.504
	PAM	Baseline	248	0.643
		Pre-inoculation	213	0.735
		Post-inoculation	823	0.574
	Poly	Baseline	246	0.657
		Pre-inoculation	181	0.614
		Post-inoculation	865	0.882

^1^ SCFP—NutriTek^®^ (Diamond V, Cedar Rapids, IA, USA). ^2^ Stimulants—mock (negative control); LPS (lipopolysaccharide; mimics Gram-negative); PAM (Pam3CSK4; mimics Gram-positive); Poly (mixture of Poly(I:C) and imiquimod; mimics virus). ^3^ Timepoint—baseline (week 1); pre-inoculation (week 7); post-inoculation (weeks 10 and 13).

**Table 6:** Linear regression model ^1^ of PAM ^2^-induced pro-inflammatory cytokine IL-6 production by innate cells from the blood of steers supplemented with or without *Saccharomyces cerevisiae* fermentation products (SCFPs ^3^) with fixed effects only when compared to values from the mock assay. n = 167.

	Estimate	Standard Error	t-Value	*p*-Value
**Intercept**	**−4.008**	**1.782**	**−2.249**	**0.026**
Control = ref. level				
SCFP supplementation	−0.038	0.108	−0.356	0.722
Baseline timepoint = ref. level				
Pre-inoculation timepoint	0.189	0.136	1.388	0.167
Post-inoculation timepoint	−0.348	0.505	−0.689	0.492
**Bodyweight (kg)**	**0.832**	**0.348**	**2.390**	**0.018**
M-stage M0 = ref. level				
M-stage M1	0.149	0.519	0.287	0.775
M-stage M2	−0.023	0.503	−0.045	0.964
M-stage M4/M4.1	−0.172	0.527	−0.327	0.744
M-stage M4P/M4.1P	0.185	0.528	0.351	0.726
SCFP supplementation*M-stage M1	−0.064	0.245	−0.263	0.793
SCFP supplementation*M-stage M2	0.048	0.198	0.241	0.810
SCFP supplementation*M-stage M4/M4.1	**0.604**	**0.283**	**2.138**	**0.034**
SCFP supplementation*M-stage M4P/M4.1P	0.200	0.293	0.684	0.495

^1^ IL-6 response to PAM ~ Group + Timepoint + log_BW + Group*M-stage. ^2^ Stimulant—PAM (Pam3CSK4; mimics Gram-positive exposure). ^3^ SCFP—NutriTek^®^ (Diamond V, Cedar Rapids, IA, USA).

**Table 7:** Linear mixed regression model ^1^ of LPS ^2^-induced pro-inflammatory cytokine IL-1β production by innate cells from the blood of steers supplemented with or without *Saccharomyces cerevisiae* fermentation products (SCFPs ^3^) with steer ID fitted as a random effect when compared to values from the mock assay. n = 167.

	Estimate	Standard Error	t-Value	*p*-Value
Intercept	0.408	0.859	0.476	0.635
Control = ref. level				
**SCFP supplementation**	**−0.135**	**0.048**	**−2.840**	**0.005**
Baseline timepoint = ref. level				
Pre-inoculation timepoint	−0.024	0.054	−0.437	0.663
Post-inoculation timepoint	−0.250	0.200	−1.248	0.214
Bodyweight (kg)	−0.001	0.167	−0.003	0.998
M-stage M0 = ref. level				
M-stage M1	0.165	0.203	0.815	0.417
M-stage M2	0.088	0.191	0.463	0.644
M-stage M4/M4.1	0.015	0.202	0.076	0.939
M-stage M4P/M4.1P	0.162	0.203	0.801	0.425
SCFP supplementation*M-stage M1	0.001	0.096	0.008	0.993
SCFP supplementation*M-stage M2	**0.155**	**0.072**	**2.145**	**0.034**
SCFP supplementation*M-stage M4/M4.1	**0.217**	**0.103**	**2.098**	**0.038**
SCFP supplementation*M-stage M4P/M4.1P	0.150	0.109	1.375	0.172

^1^ IL-1β response to LPS ~ Group + Timepoint + log_BW + Group*M-stage + 1|ID. ^2^ Stimulant—LPS (lipopolysaccharide; mimics Gram-negative exposure). ^3^ SCFP—NutriTek^®^ (Diamond V, Cedar Rapids, IA, USA).

**Table 8:** Linear mixed regression model ^1^ of Poly ^2^-induced pro-inflammatory cytokine IL-1β production by innate cells from the blood of steers supplemented with or without *Saccharomyces cerevisiae* fermentation products (SCFPs ^3^) with steer ID fitted as a random effect when compared to values from the mock assay. n = 167.

	Estimate	Standard Error	t-Value	*p*-Value
Intercept	0.598	1.801	0.332	0.740
Control = ref. level				
SCFP supplementation	−0.081	0.086	−0.942	0.349
Baseline timepoint = ref. level				
**Pre-inoculation timepoint**	**−0.292**	**0.130**	**−2.243**	**0.026**
**Post-inoculation timepoint**	**0.375**	**0.160**	**2.334**	**0.021**
Bodyweight (kg)	0.158	0.352	0.450	0.654

^1^ IL-1β response to Poly ~ Group + Timepoint + log_BW + 1|ID. ^2^ Stimulant—Poly (mixture of Poly(I:C) and imiquimod; mimics virus exposure). ^3^ SCFP—NutriTek^®^ (Diamond V, Cedar Rapids, IA, USA).

**Table 9:** Linear mixed regression model ^1^ of PAM ^2^-induced pro-inflammatory cytokine IL-6 production by innate cells from the blood of steers supplemented with or without *Saccharomyces cerevisiae* fermentation products (SCFPs ^3^) with steer ID fitted as a random effect when compared to values from the mock assay. n = 167.

	Estimate	Standard Error	t-Value	*p*-Value
Intercept	−3.582	1.958	−1.830	0.070
Control = ref. level				
SCFP supplementation	−0.084	0.111	−0.763	0.447
Baseline timepoint = ref. level				
Pre-inoculation timepoint	0.236	0.131	1.803	0.073
Post-inoculation timepoint	−0.329	0.486	−0.676	0.500
Bodyweight (kg)	0.749	0.382	1.962	0.052
M-stage M0 = ref. level				
M-stage M1	0.154	0.496	0.310	0.757
M-stage M2	−0.054	0.470	−0.114	0.909
M-stage M4/M4.1	−0.145	0.498	−0.292	0.770
M-stage M4P/M4.1P	0.200	0.499	0.401	0.689
SCFP supplementation*M-stage M1	−0.018	0.234	−0.079	0.937
SCFP supplementation*M-stage M2	0.192	0.179	1.070	0.287
SCFP supplementation*M-stage M4/M4.1	**0.723**	**0.258**	**2.804**	**0.006**
SCFP supplementation*M-stage M4P/M4.1P	0.256	0.270	0.948	0.345

^1^ IL-6 response to PAM ~ Group + Timepoint + log_BW + Group*M-stage + 1|ID. ^2^ Stimulant—PAM (Pam3CSK4; mimics Gram-positive exposure). ^3^ SCFP—NutriTek^®^ (Diamond V, Cedar Rapids, IA, USA).

**Table 10:** Linear mixed regression model ^1^ of Poly ^2^-induced pro-inflammatory cytokine IL-6 production by innate cells from the blood of steers supplemented with or without *Saccharomyces cerevisiae* fermentation products (SCFPs ^3^) with steer ID fitted as a random effect when compared to values from the mock assay. n = 167.

	Estimate	Standard Error	t-Value	*p*-Value
Intercept	−4.221	1.950	−2.165	0.033
Control = ref. level				
SCFP supplementation	−0.022	0.092	−0.239	0.812
Baseline timepoint = ref. level				
Pre-inoculation timepoint	−0.010	0.146	−0.071	0.944
Post-inoculation timepoint	0.290	0.177	1.635	0.104
**Bodyweight (kg)**	**0.844**	**0.381**	**2.215**	**0.029**

^1^ IL-6 response to Poly ~ Group + Timepoint + log_BW + 1|ID. ^2^ Stimulant—Poly (mixture of Poly(I:C) and imiquimod; mimics virus exposure). ^3^ SCFP—NutriTek^®^ (Diamond V, Cedar Rapids, IA, USA).

## Data Availability

The dataset presented in this article is not readily available, because it belongs to a commercial entity. Requests to access the datasets should be directed to Dr. Ilkyu Yoon at iyoon@diamondv.com.
